# Nemesia Root Hair Response to Paper Pulp Substrate for Micropropagation

**DOI:** 10.1100/2012/859243

**Published:** 2012-01-04

**Authors:** Pascal Labrousse, David Delmail, Raphaël Decou, Michel Carlué, Sabine Lhernould, Pierre Krausz

**Affiliations:** ^1^Groupement de Recherche Eau, Sol, Environnement (GRESE EA4330), Laboratoire de Botanique et Cryptogamie, Faculté de Pharmacie, Université de Limoges, 2 rue du Docteur Marcland, 87025 Limoges Cedex, France; ^2^Laboratoire de Chimie des Substances Naturelles (LCSN EA 1069), Faculté des Sciences et Techniques, Université de Limoges, 123 Avenue Albert Thomas, 87060 Limoges Cedex, France; ^3^Laboratoire de Pharmacognosie et de Mycologie, UMR CNRS 6226 SCR, Université de Rennes 1, Equipe PNSCM, 2 Avenue du Professeur Léon Bernard, 35043 Rennes Cedex, France

## Abstract

Agar substrates for *in vitro* culture are well adapted to plant micropropagation, but not to plant rooting and acclimatization. Conversely, paper-pulp-based substrates appear as potentially well adapted for *in vitro* culture and functional root production. To reinforce this hypothesis, this study compares *in vitro* development of nemesia on several substrates. Strong differences between nemesia roots growing in agar or in paper-pulp substrates were evidenced through scanning electron microscopy. Roots developed in agar have shorter hairs, larger rhizodermal cells, and less organized root caps than those growing on paper pulp. In conclusion, it should be noted that in this study, *in vitro* microporous substrates such as paper pulp lead to the production of similar root hairs to those found in greenhouse peat substrates. Consequently, if agar could be used for micropropagation, rooting, and plant acclimatization, enhancement could be achieved if rooting stage was performed on micro-porous substrates such as paper pulp.

## 1. Introduction

Micropropagation is a powerful biotechnology for plant multiplication [1, 2], but plant losses during acclimatization in greenhouse reduced, for some species, the asset of *in vitro* culture multiplication. *In vitro *rooting induction can be mediated by adding plant growth regulators or hormone-like substances to the culture medium [3]. However, the survival rate of these plants during acclimatization is low [3, 4]. In fact, greenhouse culture conditions like hygrometry, CO_2_ levels, and nutrient bioavailability in culture medium are drastically different from those used for *in vitro* micropropagation. Most of the time, *in vitro* culture medium is composed of macro- and micronutrients, vitamins, carbohydrates, and eventually plant growth regulators gelified by polysaccharidic substances like agar. So, root formation *in vitro* could be drastically different from in classical greenhouse substrates. Gonçalves et al. [3] suggested that the lower survival rate during plant acclimatization is due to nonfunctionality of the *in vitro* developed rooting system. Root hairs constitute the major plant/substrate interface as they represent as much as 70% of the plant root surface [5, 6]. So, it could be assumed that root-hair nonfunctionality can drastically reduce water and mineral nutrient uptake, thus representing a limiting key step to acclimatization in peat substrate. 

As first proposed by Afreen-Zobayed et al. [4] for sweet potato, paper pulp could be a potentially suitable substrate for *in vitro* culture and functional root-hair production. In order to clarify this assumption, this study compares *in vitro* development of an ornamental plant, *Nemesia denticulata *(Scrophulariaceae), on several substrates like agar and paper pulp. Moreover, enhancement of nemesia acclimatization through the use of paper-pulp substrate was evaluated.

## 2. Experimental Procedures

### 2.1. Preparation of Paper-Pulp Miniplugs

Paper pulp (a mixture of wood fibers from deciduous trees) was kindly provided by L. Harvengt from AFOCEL (http://www.fcba.fr/). Paper pulp was rehydrated in boiling water (200 g dry mass·L^−1^) for 30 min and then vigorously mixed during 30 min in order to eliminate remaining aggregates. After supplemental water draining, paper pulp was manually pressed in plug molds (16 × 15 mm, *Ø* × *H*) and dried at 50°C for 24 h.

### 2.2. Plant Culture and Acclimatization

*Nemesia denticulata *(Scrophulariaceae) plants were cultivated on Murashige and Skoog's (MS) modified by Van der Salm et al. [7] medium supplemented with 20 g·L^−1^ sucrose and 7 g·L^−1^ agar HP-696 (Kalys). The pH was adjusted to 5.8 before autoclaving at 121°C (106 kPa) for 20 min. Cultures were maintained at 22 ± 2°C under fluorescent lights (20 *μ*mol·m^−2^·s^−1^ of PAR light (photosynthetically active radiation), photoperiod 16 h/24 h) (Grolux 36W). After 3 subcultures, plants were placed on 4 different rooting substrates: agar 7 g·L^−1^ (A), paper pulp prepared as miniplugs (PP), sorbarod (S) (cellulose plugs from Baumgartner Papiers), and peat (fertil miniplug) (P) as control. All substrates were supplemented with 5 mL of liquid half-strength MS Van der Salm medium. After 25 days of *in vitro *culture, the plants were divided into 2 batches: 24 plants per treatment were harvested and 24 other plants per treatment were then transferred to greenhouse for 21 days of acclimatization under fog (cycle of 3 min per hour, 4 times per day during 7 days). Root and shoot fresh and dry masses were measured.

### 2.3. Scanning Electron Microscopy

For scanning electron microscopy, 2-cm-long root tips from the apex were dehydrated in an ethanol-graded series (10 min at 10°, 10 min at 30°, 10 min at 50°, 10 min at 70°, 10 min at 90°, and three 15 min times at 100°). After critical point drying with CO_2_ (FL9496 critical point dryer, Balzers Union), samples were mounted on stubs and coated with 17 nm of gold/paladium (SCD050 sputter coater, Baltec). Root observations were realized using a Philips XL30 scanning electron microscope at 10 kV. Root hair length was measured using Visilog Viewer 6.820 (Noesis).

### 2.4. Data Analysis

The data were analyzed using R.2.9.2 software. For all further statistical tests, the null hypothesis was the data normality or homogeneity, and the alpha level was set at 0.05 (data are nonnormal or heterogeneous when *P* value <0.05).

Normality of the measurement data matrix for culture was tested with multivariate Shapiro-Francia test [8] which indicates that the results are not normally distributed (*P* = 2.649*e* − 05). Thus, only nonparametric tests will be used to process the matrix.

The experiment was laid down in a randomized complete block design. Thus, for each treatment, experiments were carried out with 24 plants and repeated in duplicate. As the data distribution was not normal, the nonparametric ANOVA Friedman test [9] was used to check if duplicates were homogenous, and no difference between duplicates was evidenced. The Friedman test [9] adapted to plant data [10] and the nonlinear principal component analysis [11] were used for medium comparisons.

## 3. Results

### 3.1. Scanning Electron Microscopy

**Figure 1:**
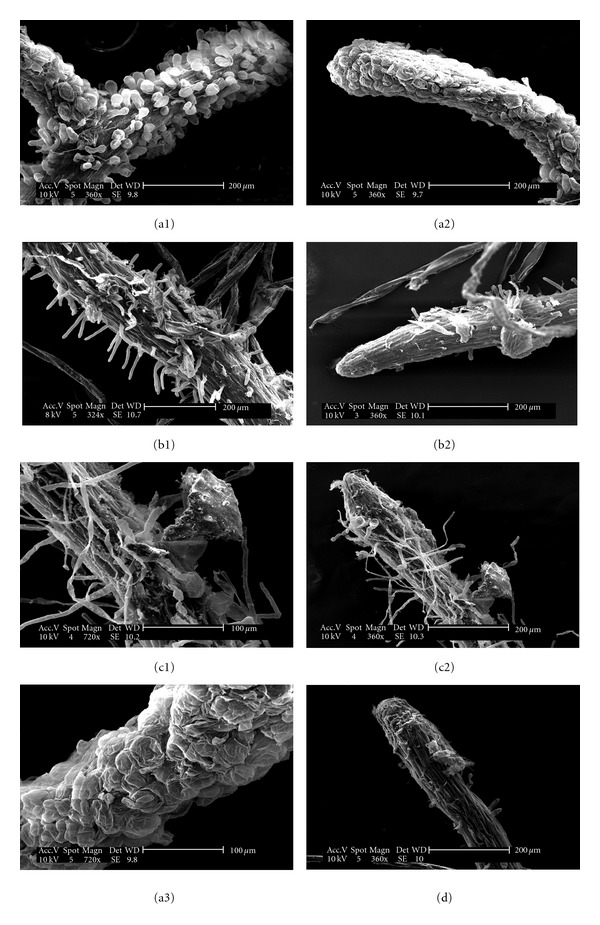
Nemesia roots cultivated on agar (a), paper pulp (b), and control plant (c) from greenhouse. (a1) Root hair of nemesia plant cultivated on agar medium. Note the strongly modified cellular morphology even at higher magnification in (a3). (a2) Root cap of nemesia cultivated in agar medium. No root cap is clearly identifiable. (b1) Root hair structure in nemesia root cultivated on paper pulp. (b2) Root cap of nemesia cultivated on paper pulp. Note the quiet similar structure to the control plant from greenhouse (c2). (c1) Root hair structure of the nemesia root cultivated in peat in greenhouse conditions. (c2) Root cap of nemesia cultivated in greenhouse conditions in peat. (d) Root cap of nemesia cultivated in sorbarod.

Major differences in root-hair morphology and length between plants growing on agar and on paper-pulp substrate (Figures 1(a) and 1(b), resp., Figure 4) were evidenced whereas roots from paper pulp and sorbarod were quite similar (Figures 1(b) and 1(d), Figure 4). It should be noted that root hairs were drastically shortened on agar in comparison with those obtained on paper-pulp substrate (Figures 1(a1), 1(a3), and 1(b1), resp., Figure 4). Moreover, root apex (epidermal cells and cap) strongly differed between the two treatments. Root cap in agar was less organized and epidermal cells were inflated and ovoid (Figure 1(a2)), whereas roots from plants cultivated on paper-pulp substrate (Figure 1(b)) presented a quiet similar morphology to roots from control plants (roots growing in greenhouse on peat substrate) which exhibited long root hairs (Figure 1(c1), Figure 4) and highly organized root cap (Figure 1(c2)).

### 3.2. Plant Biomass and Water Content

**Figure 2:**
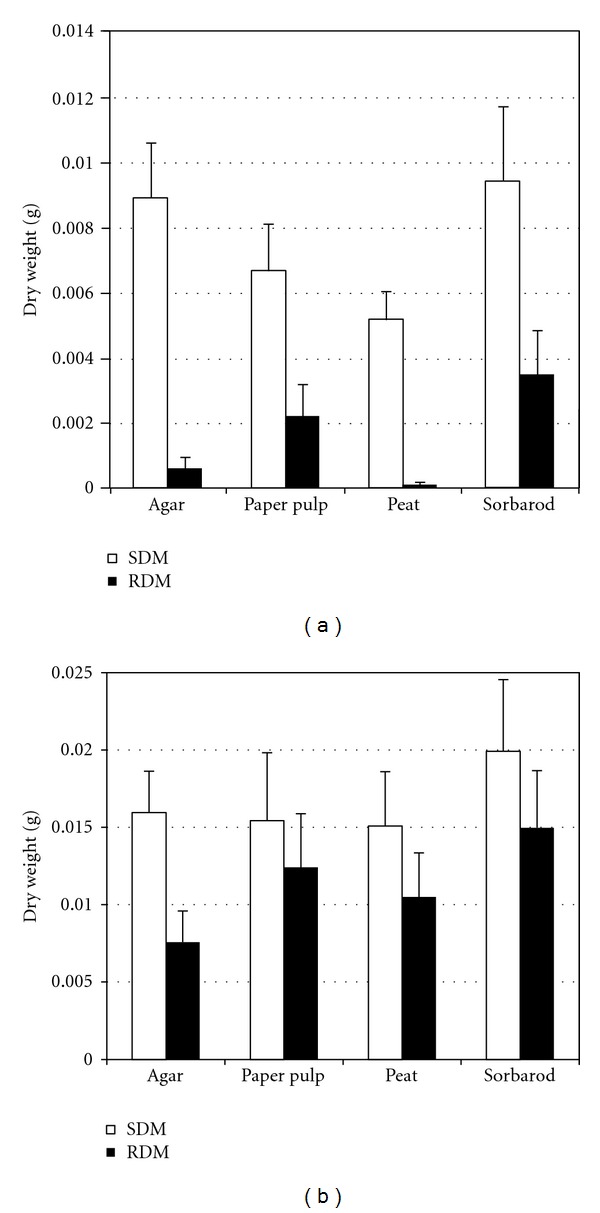
Dry mass of nemesia after 25 days of *in vitro* culture (a) and after 21 days of acclimatization (b). SFM: shoot fresh mass, RFM: root fresh mass, SDM: shoot dry mass, and RDM: root dry mass. Data are mean ± se, *n* = 24.

**Figure 3:**
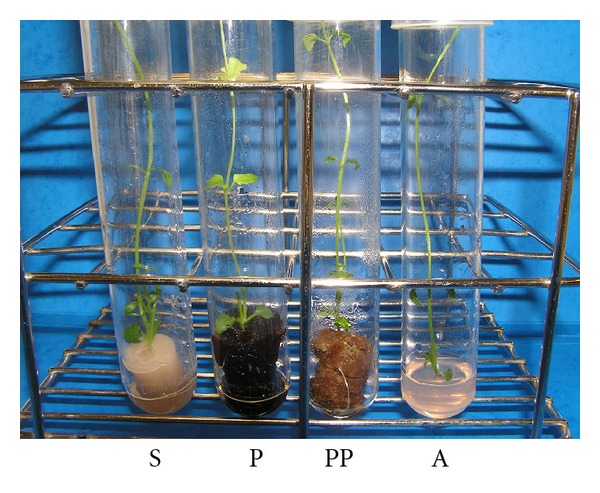
Aspect of nemesia plants on the different substrates during *in vitro* culture phase. S: sorbarod; P: peat; PP: paper pulp; A: agar.

**Figure 4:**
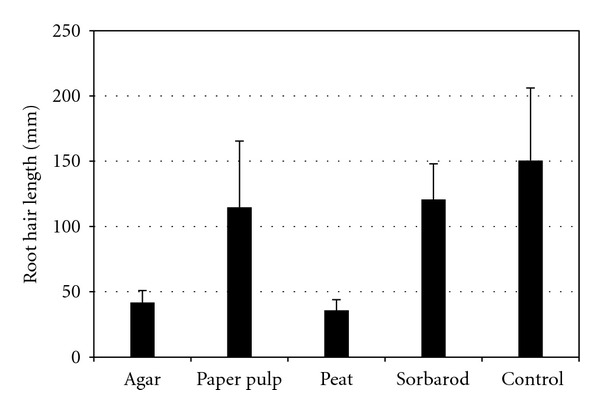
Root hair length in the different substrates *in vitro*. Control: root hair of the nemesia cultivated in peat in greenhouse conditions. Data are mean ± s.e., *n* = 30.

**Figure 5:**
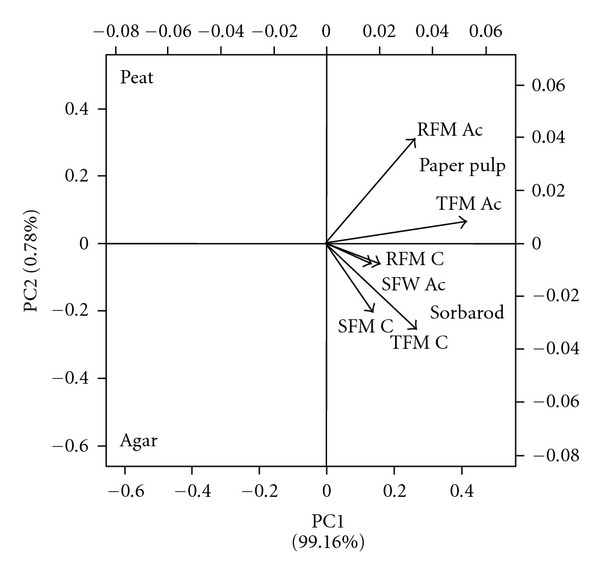
Biplot of nonlinear principal component analysis of *in vitro* cultured and acclimatized nemesia in the four substrates. RFM Ac: root fresh mass of acclimatized nemesia; RFM C: root fresh mass of *in vitro* cultured nemesia; SFM Ac: shoot fresh mass of acclimatized nemesia; SFM C: shoot fresh mass of *in vitro* culture nemesia; TFM Ac: total fresh mass of acclimatized nemesia; TFM C: total fresh mass of *in vitro* cultured nemesia. N.B.: dry masses are not visible due to high clustering near the origin.

Plant biomass and water content were determined before and after 21 days of acclimatization (Figures 2(a) and 2(b) and Table 1). During the *in vitro* culture phase, no significant differences were observed between the paper pulp and agar (*P* = 1.000) and between agar and sorbarod (*P* = 0.317) even if sorbarod appeared as the best substrate for this stage in terms of biomass production (Figures 2(a) and 3). All the substrates appeared more potent for this micropropagation phase than peat (*P* = 0.046). For the acclimatization stage (Figure 2(b)), sorbarod differed from agar (*P* = 4.678*e* − 3) and peat (*P* = 0.034) but not from paper pulp (*P* = 0.479) which differed from peat (*P* = 4.678*e* − 3). Nonlinear principal component analysis (Figure 5) evidenced that paper-pulp-based substrates were the best for *in vitro* culture and acclimatization phase of nemesia. It should be noted that the two paper-pulp based substrates were very similar as the PC2 axis contributes only to 0.78% of the discrimination. Contrariwise, PC1 axis, contributing to 99.16% of the discrimination, clearly segregates these two media from peat and agar. Root fresh and dry masses of *in vitro* plants contributed, respectively, to 13.02% and 11.30% of the discrimination along the PC1 axis (Table 2). Moreover, shoot fresh mass and root dry mass of acclimatized plant contributed to 11.33% and 10.43%, respectively. Along the PC2 axis, the main discriminant was the shoot fresh mass of the acclimatized plants (11.56%). It could be noted that root dry mass of *in vitro* cultured and acclimatized plants contributed to 6.91% and 8.10% of the discrimination, respectively. For the plant water content, no significant difference could be observed between all substrates during the acclimatization phase. A slight increase in water content was evidenced in roots of *in vitro* nemesia from agar and peat but not in shoots.

## 4. Discussion

Roots of nemesia cultivated on agar medium have similar phenotype to hairless root mutants [6]. Absences of root hair and poor growth are attributed by several authors to hypoxia in agar medium [12, 13]. In addition, Bidel et al. [14] reported that root meristems emerging from the agar gel thereafter progressed quicker than meristems remaining in the gel. These authors hypothesized the presence of several limiting factors for root growth in agar medium in addition of O_2_ depletion: progressive dehydration, acidification, and mineral depletion around the older root segments may also have reduced the meristem growth. Moreover, actively tip-growing root hair cells are characterized by a polarized apex rich in Golgi vesicles and mitochondria [15] suggesting important ATP needs for root-hair growth. High amounts of ATP in root hair imply a good O_2_ pressure in the substrate [14]. The diffusion of O_2_ in agar medium is lower than those found in conventional substrates. In fact, substrates other than agar, including sorbarod [16–19], foam [20–22], vermiculite [23], a vermiculite/gelrite mixture [24], peat [25], rockwool [26], coir [27], and a paper-pulp/vermiculite mixture [4], have been used to prevent low O_2_ pressure and poor rooting in agar medium. Decreased O_2_ level in a medium could be directly associated with a decrease in root-hair length and to a complementary extent with a decrease in root respiration [14]. This could result from the direct effect of redox state on gene expression as Sánchez-Fernández et al. [28] demonstrated that the redox state of cellular thiols plays a key role in root-hair growth (for an update see [29, 30]). Consequences are a decrease in water, nutrient uptake, and biomass production. In a controlled and confined environment like a culture tube, plants growing on agar medium do not suffer from this poorly functional rooting system and absorb water and nutrients directly through epidermal and/or rhizodermal cells. But in greenhouse environment, atmospheric water amount is limited, and roots must assume the water and nutrient supply. Even under fog, more than 21 days of culture were necessary for tending towards a complete restoration of physiological processes. Then, the nonfunctionality of the *in vitro* rooting system developed in agar has no consequence on *in vitro* plants but has deleterious effects on plant acclimatization in greenhouse (for a review, see Hazarika [31]). 

On the other hand, cheap alternatives to agar for micropropagation are currently under research from low-cost gelling agent to vegetables fibers or vegetables byproducts like Isabgol [32–35], sugarcane bagasse [36, 37], plant gums [38–40], plant fibers [41–43], starch [44, 45], or other systems devoided of agar [46–52]; for a review, see Gangopadhyay et al. [53]. In this way, paper pulp could be evaluated alone or in association with compounds leading to enhance the porosity of the substrate. Similarly, Barrett-Lennard and Dracup [12] demonstrated that plant growth was increased even in porous agar-gelled media. Cellulose plugs like sorbarod constitute a good alternative for agar-gelled media but in the sorbarod system, plant roots pass through the pore of the plug, and only few ramifications were produced. Moreover, roots grown on filter paper matrix were often problematic to take out without injury. Paper-pulp plugs with enhanced porous structure could combine the advantages of sorbarod with a well-ramified rooting system like these obtained in paper-pulp experiments.

In that sense paper, pulp appears as a good alternative to agar for rooting *in vitro* cultured plants before acclimatization even if a best aeration of paper-pulp miniplugs should be achieved in order to enhance the rooting-system development.

## Figures and Tables

**Table 1:** Nemesia water content (%) after 25 days of *in vitro* culture and after 21 days of acclimatization in greenhouse conditions.

Water content (%)	*In vitro* culture		Acclimatization	
	Shoot	Root	Shoot	Root
Agar	90.6 ± 1.41	95.36 ± 2.40	89.45 ± 0.84	90.81 ± 1.38
Paper pulp	92.06 ± 0.61	88.90 ± 1.90	88.97 ± 0.75	90.74 ± 1.22
Peat	91.36 ± 2.15	98.54 ± 0.24	87.94 ± 0.79	90.05 ± 0.99
Sorbarod	91.95 ± 0.65	92.36 ± 2.46	88.22 ± 0.76	89.18 ± 0.82

**Table 2:** Contribution percentage of each variable to discrimination between the four substrates along PC1 and PC2 axis in the nonlinear principal component analysis. RDM Ac: root dry mass of acclimatized nemesia; RDM C: root dry mass of *in vitro* cultured nemesia; RFM Ac: root fresh mass of acclimatized nemesia; RFM C: root fresh mass of *in vitro* cultured nemesia; SDM Ac: shoot dry mass of acclimatized nemesia; SDM C: shoot dry mass of *in vitro* cultured nemesia; SFM Ac: shoot fresh mass of acclimatized nemesia; SFM C: Shoot fresh mass of *in vitro* culture nemesia; TFM Ac: Total fresh mass of acclimatized nemesia; TFM C: total fresh mass of *in vitro* cultured nemesia; TDM Ac: total dry mass of acclimatized nemesia; TDM C: total dry mass of *in vitro* cultured nemesia.

Variable	Contribution along PC1 axis	Contribution along PC2 axis
SFM C	0.1%	8.23%
SFM Ac	11.33%	11.56%
SDM C	2.20%	4.99%
SDM Ac	1.93%	4.70%
RFM C	13.02%	5.19%
RFM Ac	7.83%	0.05%
RDM C	11.29%	6.91%
RDM Ac	10.43%	8.10%
TFM C	0.11%	6.57%
TFM Ac	9.65%	0.04%
TDM C	2.28%	4.39%
TDM Ac	1.38%	5.11%

## Abbreviations

MS: Murashige and Skoog medium.
